# Influence of Waveform Characteristics on LiDAR Ranging Accuracy and Precision

**DOI:** 10.3390/s18041156

**Published:** 2018-04-10

**Authors:** Xiaolu Li, Bingwei Yang, Xinhao Xie, Duan Li, Lijun Xu

**Affiliations:** 1 School of Instrumentation Science and Opto-Electronics Engineering, Beihang University, Beijing 100191, China; xiaoluli@buaa.edu.cn (X.L.); bingweiyang1966@163.com (B.Y.); xinhaoxie@buaa.edu.cn (X.X.); duanli@buaa.edu.cn (D.L.); 2 Beijing Advanced Innovation Center for Big Data-Based Precision Medicine, Beijing 100191, China

**Keywords:** LiDAR ranging, accuracy, precision, waveform characteristic

## Abstract

Time of flight (TOF) based light detection and ranging (LiDAR) is a technology for calculating distance between start/stop signals of time of flight. In lab-built LiDAR, two ranging systems for measuring flying time between start/stop signals include time-to-digital converter (TDC) that counts time between trigger signals and analog-to-digital converter (ADC) that processes the sampled start/stop pulses waveform for time estimation. We study the influence of waveform characteristics on range accuracy and precision of two kinds of ranging system. Comparing waveform based ranging (WR) with analog discrete return system based ranging (AR), a peak detection method (WR-PK) shows the best ranging performance because of less execution time, high ranging accuracy, and stable precision. Based on a novel statistic mathematical method maximal information coefficient (MIC), WR-PK precision has a high linear relationship with the received pulse width standard deviation. Thus keeping the received pulse width of measuring a constant distance as stable as possible can improve ranging precision.

## 1. Introduction

Time of flight (TOF) based light detection and ranging (LiDAR) is a technology for measuring the distance between the laser transmitter and the target [1,2,3]. Combining the measured distance with the laser beam steering, a three-dimensional (3D) point cloud of the target is generated in an arbitrary Cartesian mapping system [4]. The accuracy of point cloud is governed by three main errors, caused by the direct geo-referencing of laser beam, the measured range of laser itself and the mounting and scanning geometry [5]. The error derived from the measured range of laser is a basic and crucial issue, which is directly related to the trigger signals (start/stop signals) for calculating the time of flight. The most common ways of time interval measurements are divided into two categories, including time-to-digital converter (TDC) based measurement that counts time between trigger signals and analog-to-digital converter (ADC) based measurement that estimates the time interval between start/stop signals with appropriate algorithm [6]. Since TDC based LiDAR and ADC based LiDAR are both related to the echo pulse characteristics, it is very important for two kinds of ranging systems to study the influence of waveform characteristics on range measurement accuracy and precision.

TDC based LiDAR mainly solves the distance measurement problems of concerning the ranging accuracy in the analog domain, which requires high performance analog discrete return system with fast rise time pulses and high receive bandwidth [7,8]. Due to uncertain reflectivity of the target surface and noise interference, the performance of analog discrete return systems cannot meet the requirement of the input signal, which results in shape variation of echo waveform, and consequently leads to worse ranging accuracy and precision. In order to improve ranging accuracy and precision, walk error and timing jitter need to be eliminated, which are the main errors in the analog discrete return systems [9,10]. As the main systematic error, walk error is caused by variation of amplitude and shape of input pulse [9,10]. Timing jitter refers to the timing deviation caused by statistical fluctuations existing in shape of the detected pulses, which is the largest random error [7,10]. A TDC based circuit combining constant fraction discriminator (CFD) is usually used reduce the walk error due to its insensitivity to pulse amplitude. However, because of increased input overdrive of the comparator, extra-walk-error can be generated by the comparator of CFD. In addition, the distorted signal due to detector saturation also introduces error at a short detection distance, which results in an inaccurate time discriminator. Thus, the walk error and the timing jitter are not reduced adequately in analog discrete return systems, especially when the input pulse amplitude is varied in a wide dynamic range [11].

A high-speed ADC based LiDAR has a number of performance advantages when using a waveform-digitizing system over those based on TDC [6,12]. Because the ADC digitizes the return signal in the digital domain (instead of simply measuring time), waveform processing can be employed to implement sophisticated detection schemes that not only have better performance than the TDC, but also provide additional information for target identification [6,12]. The digitized signal is sent to a digital signal processor (DSP) or host computer, which will execute a appropriate algorithm to analyze the digitized waveform to estimate the location of the target in the return signal [13,14]. The range measurement accuracy is directly related to the ADC sampling frequency and time discriminator algorithms. Currently, three kinds of algorithms are commonly used [15]. Leading edge discrimination is a very simple method. Yet, it is limited by the noise in the environment and the amplitude variation. Peak estimation method calculates the time interval between peaks, which is directly related to the echo shape and resolution of the ADC used in the system. Cross correlation method calculates the highest correlation value between the Gaussian model and reference signal. Because the time discriminator algorithms are based on the waveform recorded by high-speed ADC, the accuracy of time discriminator will be influenced by the shape of echo pulse [16].

In order to research the influence of waveform characteristics on the ranging accuracy and precision, two lab-built LiDAR (TDC based analog discrete return system and ADC based waveform-digitizing system) are designed to collect the measured data (e.g., time instance, digitized waveform) and waveform characteristics (e.g., amplitude, SNR, pulse width StD, etc.) are extracted from the digitized waveform. Five time discriminator algorithms used for processing digitized waveforms are compared according to their ranging accuracy, precision, and execution time. Then, given the same input signal, the ranging accuracy and precision results of the ADC based waveform-digitizing system (using the aforementioned algorithms) and TDC-based analog discrete return system are compared to demonstrate the influence of waveform characteristics. Finally, the relationships of waveform characteristics and the ranging accuracy and precision are analyzed using a statistic method maximal information coefficient (MIC) [17]. The findings can be used to LiDAR system design and error modeling.

The rest of this paper is organized as follows. Section 2 describes the lab-built LiDAR system, including analog discrete return system and waveform-digitizing system. The time discriminator algorithms for processing digitized signal recorded by the waveform-digitizing system are elaborated in Section 3. In Section 4, experiments are performed to demonstrate the influence of waveform characteristics on the ranging accuracy and precision. Finally, conclusions are presented in Section 5.

## 2. System

### 2.1. Lab-Built LiDAR System

**Figure 1:**
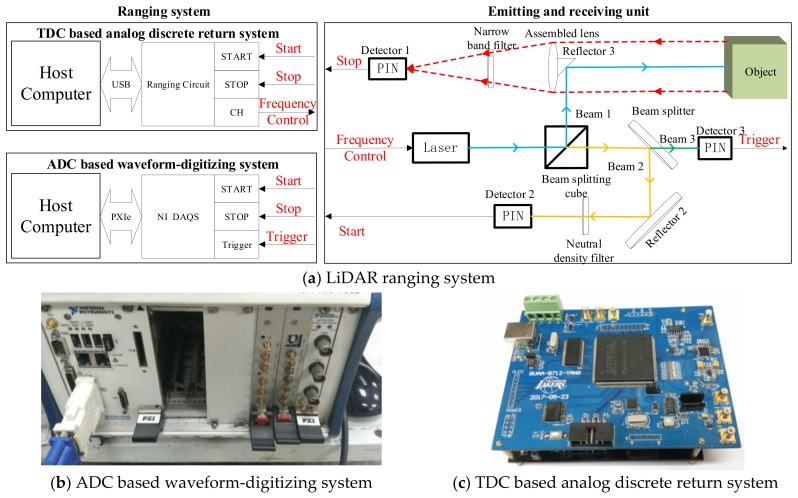
Two kinds of LiDAR ranging system.

In our lab, LiDAR was built consisting of the emitting and receiving unit and the range measurement system. In order to obtain the accurate time of the emitted and received pulses, two types of ranging system were designed to estimate the range: one is TDC based analog discrete return system of auto gain control (AGC) and constant fraction discriminator (CFD) [18], the second one is ADC based waveform-digitizing system for waveform-post-processing [19]. As shown in Figure 1, these two types of ranging systems share the same emitting and receiving unit and obtain the measured data for researching the influencing factors of ranging accuracy and precision.

In the emitting and receiving unit, a solid state Q-switched Nd:YAG laser is employed as laser emitter, yielding pulses of 10 ns duration and 1064 nm wavelength. As shown in Figure 1, the emitted pulses are split into three pulses (Beam 1, Beam 2, and Beam 3) by beam splitting cube and beam splitter. One of the pulses (Beam 1) is the emitted pulse to the target, which is reflected by the reflector (Reflector 3) of coaxial lens. The echo from the object is gathered by the assembled lens with a diameter of 35 mm and then is converted into electronic signal (Stop) by the PIN detector (Detector 1). The second pulse (Beam 3) is converted into electronic signal (Trigger) by Detector 3. The last pulse (Beam 2) is converted into electronic signal (Start) after being reflected by Reflector 2 and filtered by neutral density filter. The start signal and stop signal are used to calculate the time interval for estimating the distance.

The first kind of ranging system is developed based on analog discrete return system for measuring the time interval by using a TDC chip with high counting resolution. In the analog discrete return system, auto gain control (AGC), and constant fraction discriminator (CFD) circuits are used to reduce the walk error and time jitter and achieve high ranging accuracy and precision. The detail specifications are presented in [18].

The second kind of ranging system is developed based on waveform-digitizing system. The data acquisition system (DAQS), as the alternative of ranging system, employs a digitizer (NI PXI-5154) to synchronously acquire the waveforms of the start and stop pulse obtained by the emitting and receiving units. The digitizer has an 8-bit dual-channel with each channel nominal synchronous sample rate 1 Gs/s (Giga sample per second). The sampled data is uploaded to the host computer through PXIe Bus. Other details are presented in [19].

### 2.2. Time Determination Based on Analog Discrete Return System

**Figure 2:**
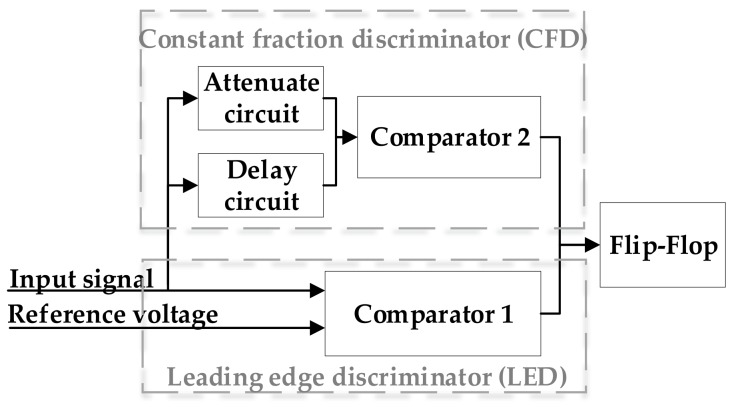
Time discriminator of constant fraction discriminator circuits.

The previous work has explained the returned amplitude changes in large dynamic range [18]. Automatic gain control (AGC) circuit is applied in analog discrete return system to stabilize the received pulse amplitude into a narrow range before being fed to the time discriminator [18]. The output of AGC is sent into constant fraction discriminator (CFD), as shown in Figure 2. In leading edge discriminator (LED) circuit, a constant threshold is set above the average noise level to avoid false trigger caused by the noise. CFD circuit consists of attenuate circuit with a parameter of the attenuation fraction and delay circuit with a parameter of the delay time. Through adjusting parameters of the attenuation fraction and the delay time, CFD circuit is designed to determine the appropriate time instance for reducing the walk error caused by the input pulse amplitude variation. The criterion of selecting parameters of the attenuation fraction and the delay time has been discussed in [18]. In this paper, the analog ranging circuit of AGC and CFD uses the attenuation fraction of 0.5 and a delay time of 2 ns.

### 2.3. Waveform-Post-Processing Based on Waveform-Digitizing System

**Figure 3:**
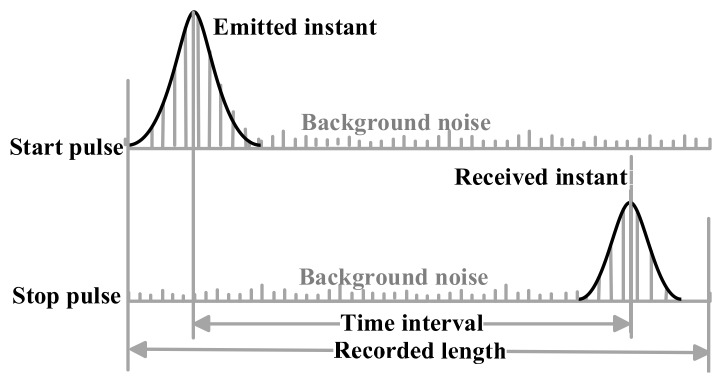
Time determination based on waveform-post-processing.

Waveform-post-processing based time determination employs the emitted and received waveform includes two steps: digitized waveform acquisition and waveform-post-processing. In the digitized waveform acquisition step, NI DAQS synchronously records the waveform of the start and stop pulse, which will be sent to the host computer through PXIe. As shown in Figure 3, the recorded waveform consisting of the pulse signal and background noise. In the waveform-post-processing step, a time discrimination method is used to extract the emitted and received time instant for calculating the distance. In order to research the influence of waveform characteristics on ranging accuracy and precision, five waveform-post-processing based time discrimination methods are introduced in Section 3.

## 3. Methodology

The influence of the circuit parameters of analog discrete return system on the ranging precision has been discussed in our previous work [18]. In this section, we elaborated five time discrimination methods for waveform-post-processing including: leading edge discriminator, peak discriminator, center of gravity discriminator, inflection discriminator (zero crossing of the second derivative), and constant fraction discriminator [20]. The illustrated methods will be discussed according to the ranging precision, accuracy, and execution time in Section 4. 

In order to estimate the time instance locating on the digitized waveform, as the literature was introduced in [21], Gaussian function is used for fitting digitized pulse waveform as

(1) $$f(t;a,b,c)=a\exp\left\{-{\left(\frac{t-b}{c}\right)}^{2}\right\}$$

where t is the flying time of the pulse, a is the amplitude of the received pulse, b is the position of the pulse peak, c indicates the pulse width. The transmitted and received times are determined based on Gaussian function using these five methods as follows.

A. Leading Edge Discriminator (LE)

**Figure 4:**
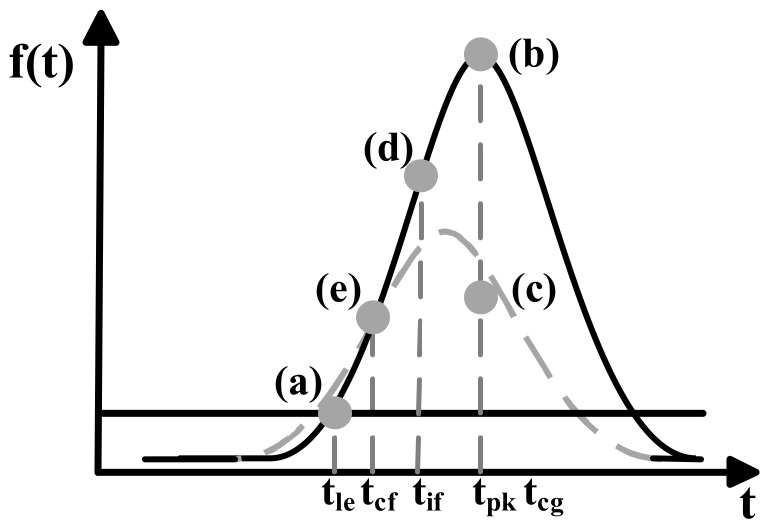
Time discrimination methods: (**a**) Leading edge, (**b**) peak, (**c**) center of gravity, (**d**) inflection, (**e**) constant fraction.

Leading edge discriminator is the most basic method for time discrimination when the leading edge exceeds a fixed threshold, which is called LE for short [22]. However, time instance determined by the LE method is strongly influenced by the pulse amplitude variation and the pulse width variation, which results in the walk error in range measurement [22]. As shown as the point (a) in Figure 4, when the leading edge of Gaussian pulse exceeds a fixed threshold $V_{th}$, the time instant ($t_{le}$) of the received pulse is written as

(2) $$t_{le}=b-c\sqrt{\ln(\frac{a}{V_{th}})}$$

B. Peak Discriminator (PK)

The peak detection method is used to estimate the maximum position of received pulse, which is called PK for short, as shown in formula (3).

(3) $$t_{pk}=t_{\max(f(t))}$$

Peak detection is insensitive to the variation of the pulse amplitude and the pulse width, but it is strongly influenced by the local spikes and the noise on the pulse waveform. As shown as the point (b) in Figure 4, the time instant ($t_{pk}$) of the received waveform is written as

(4) $$t_{pk}=b$$

C. Center of Gravity Discriminator (CG)

As shown as the point (c) in Figure 4, the center of gravity of the received waveform $t_{cg}$ is called CG for short. CG method is used to calculate energy center of the pulse waveform, which is determined from the digitized waveform integration as shown in formula

(5) $$t_{cg}=\frac{\int_{t_{1}}^{t_{2}}tf(t)dt}{\int_{t_{1}}^{t_{2}}f(t)dt}$$

where $t_{1}$ and $t_{2}$ represent boundaries on both sides of the received pulse. When $t_{1}$ and $t_{2}$ are symmetrical, the time instant ($t_{cg}$) of Gaussian function can be described by the formula (6)

(6) $$t_{cg}=b$$

CG method performs well if the echo pulse is symmetric. However, when the pulse shape is skewed distribution, the CG method based detected distance is less accurate than that using PK method [22].

D. Inflection Discriminator (IF)

Inflection discriminator method determines the time instance by calculating the zero crossing of the second derivative, which is called IF for short, as shown in formula (7).

(7) $$t_{if}=t_{\frac{\partial^{2}f(t)}{\partial t^{2}}=0,left}$$

where, $t_{\frac{\partial^{2}f(t)}{\partial t^{2}}=0,left}$ means the left point of zero crossing of the second derivative. As shown as the point (d) in Figure 4, the time instant ($t_{if}$) of the received waveform can be written as

(8) $$t_{if}=b-\frac{c}{\sqrt{2}}$$

From formula (8), it is clear that the inflection method is affected by the pulse width.

E. Constant Fraction Discriminator (CF)

As the same to theoretical definition in the analog ranging system, the method of constant fraction (called CF for short) is insensitive to the variation of input amplitude, which can reduce the walk error, but it seriously depends on the pulse shape and pulse width. As shown as the point (e) in Figure 4, the time instant ($t_{cf}$) of the received waveform is described by formula

(9) $$t_{cf}=b+\frac{c^{2}\ln k+t_{d}^{2}}{2t_{d}}$$

where k is attenuation fraction and $t_{d}$ is delay time. More details are presented for the definition of constant fraction in [18].

In summary, five kinds of time discrimination methods have various influences on ranging accuracy and precision. Thus, a series of experiments are carried out to discuss algorithm performance in Section 4.

**Figure 5:**
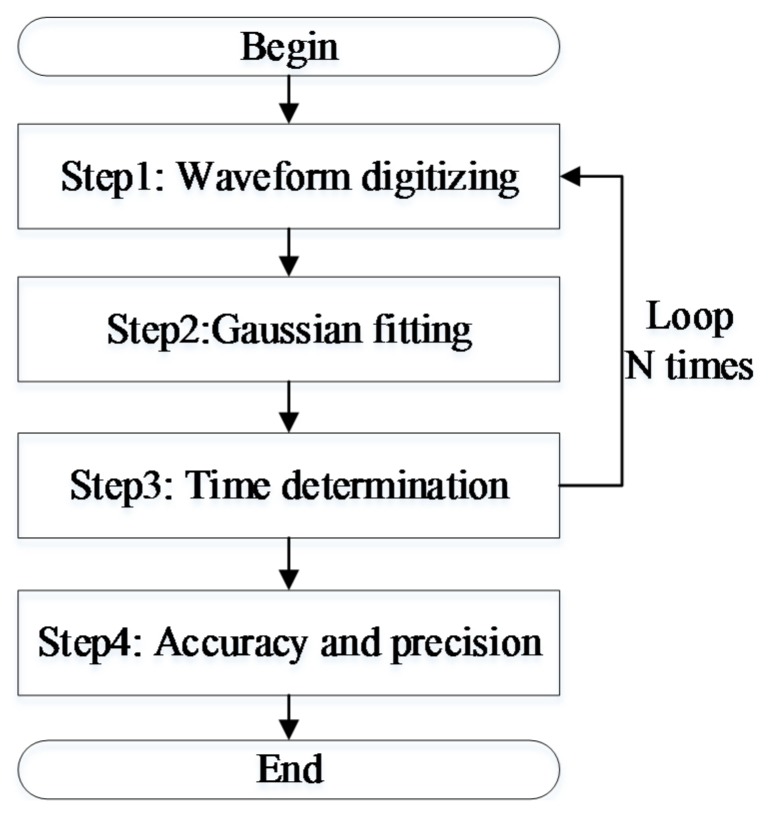
Flow chart of waveform-post-processing.

The procedure of time discrimination algorithm consists of four steps as shown in Figure 5: In step 1, the return waveform is digitized and recorded. In step 2, a Gaussian cost function is used to fit the digitized waveform using the Levenberg–Marquardt (LM) algorithm and the waveform characteristics (amplitude, position bias, and pulse width) were extracted. In step 3, the time can be determined by five aforementioned methods. The time discrimination algorithms were implemented by N times, consequently the ranging precision and accuracy of each method are estimated as the evaluation indexes in step 4. The experiment results are presented in the next section.

## 4. Experiment and Analysis

In order to research the influence of waveform characteristics on the ranging accuracy and precision, two ranging systems (analog discrete return system and waveform-digitizing system) share the same optical emitting and receiving unit to measure the time interval in different-level ranges. The relationship of waveform characteristics (SNR, amplitude, pulse width, and pulse width standard deviation (StD)) and the ranging precision and accuracy of two ranging systems were analyzed in Section 4.1, Section 4.2, Section 4.3, Section 4.4, Section 4.5 and Section 4.6. Finally, a discussion will be presented in Section 4.7.

### 4.1. Experimental Set-Up and Framework

**Figure 6:**
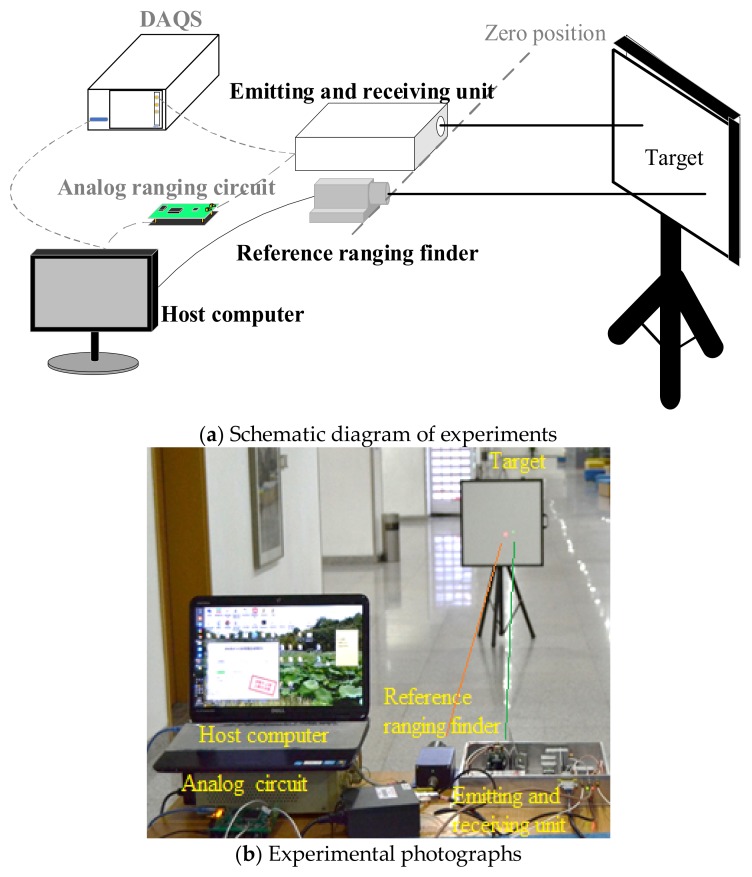
Experiments of two ranging systems and reference ranging finder.

An experimental set-up is shown in Figure 6, including host computer, analog ranging circuit, data acquisition system (DAQS), emitting and receiving unit, the reference ranging finder and the target. The commercial reference ranging finder [23] is one of the research tools for providing as the true value of the measured range, because it has a ranging accuracy of 2 mm in the range scope of 0–30 m and 3 mm in the range scope of 30–50 m. A Lambertian reflector is used as the target with a reflectance of 70%. Other devices have been described in Section 2.1. The zero position of the lab-built LiDAR system and the reference ranging finder needs to be determined before the experiments. In each experiment, the target is moved from 10 m to 46 m with a step length of 2 m, because reference ranging finder has a detection limit of 46 m. The main experimental data are stored and processed by the host computer, including the ranging data of the analog discrete return system and the reference ranging finder, and the echo pulse waveform obtained by DAQS in waveform-digitizing system. 

In Section 4.2, four key indicators including precision, accuracy, bias and nonlinearity are used to evaluate and compare the ranging performance. In Section 4.3, five waveform-post-processing based time discrimination methods are discussed according to four indicators. The methods including LE, PK, CG, IF, CF (leading edge, peak, center of gravity, inflection, and constant fraction) are used to estimate the time instance on pulse waveform. In each group data, 1000 groups of emitted and received waveform are collected to calculate one range precision, accuracy and execution time. Based on aforementioned discussion, a prior method is used to compare with the results using the analog discrete return system in Section 4.4. The ranging data obtained by the analog ranging circuit and the reference ranging finder are called AR data and RR data for short. The ranging data calculated from the waveform-post-processing based time discrimination is called WR data for short. 

At last, the influence factors of ranging accuracy and precision were analyzed in Section 4.5 and discussed in Section 4.6.

### 4.2. Definitions of the Performance Indicators

There are four main indicators to demonstrate the ranging performance including precision, accuracy, bias, and nonlinearity. The precision represents the random error of the system, while the accuracy, bias, and nonlinearity represent the systematic errors [24]. SNR (signal–noise ratio) is a widely accepted noise level parameter which is related to four former performance indicators.

Ranging precision is a quantity of how well a measurement can be made without reference to a true range value. In this manuscript, the precision represents the single-shot precision which describes the uncertainty limited by random errors in the measurement and is defined with statistical variance (σ-value) of the range measurement distribution. It is calculated from the standard deviation of measured data as formula (10).

(10) $$E_{pre}=\sqrt{\frac{1}{N}\sum_{i=1}^{N}{\left(R_{i}-\bar{R}\right)}^{2}}$$

where $E_{pre}$ is the ranging precision measured in a certain ranging position, N is the sampled number. $R_{i}$ denotes the *i*th measured data and $\bar{R}$ is the average of the measured data. 

Accuracy means the largest total error in some defined measurement range and in a defined time period. Ranging accuracy is the degree of conformity of a measured quantity to its actual value [24], which is defined by formula (11)

(11) $$E_{acu}=\frac{1}{N}\sum_{i=1}^{N}\left(R_{i}-R_{true}\right)$$

Here, $E_{acu}$ is the ranging accuracy measured in a certain ranging position, the actual (‘true’) value $R_{true}$ is obtained from the reference ranging finder, which is regarded as RR data.

Bias is the difference between average value estimated from large number of measurements and accepted true. It is equivalent to a correction to negate the systematic error that can be made by adjusting for the bias. In this manuscript, the bias is expressed by the formula

(12) $$E_{bias}=\frac{1}{M}\sum_{j=1}^{M}E_{acu,j}$$

where $E_{bias}$ is the ranging bias, M indicates the total number of ranging positions, $E_{acu,j}$ represents the ranging accuracy calculated from the *j*th ranging position.

Nonlinearity defines the deviation of the measurement results from a fitting line drawn in coordinates between the smallest and largest measured distances, which is usually given the smallest RMS (root-mean-square) deviation for the individual measurement points which is expressed by formula (13)

(13) $$E_{nonlinearity}=\sqrt{\frac{1}{M}\sum_{j=1}^{M}{\left({\bar{R}}_{j}-R_{line}\right)}^{2}}$$

where the $R_{line}$ is the fitting line drawn in coordinates between the smallest and largest measured distances of M ranging positions. ${\bar{R}}_{j}$ is the average of the measured data in *j*th ranging position.

SNR is defined as the ratio of the received amplitude and the received noise (the standard deviation of the received signal minus the fitting signal).

(14) $$SNR=\frac{a}{\sqrt{\frac{1}{O}\sum_{i=1}^{O}{\left(V_{i}-f_{i}\right)}^{2}}}$$

where a is the received amplitude, O is the number of total sampled waveform point of the received pulse, $V_{i}$ is value of the *i*th sampled point of the received signal, and $f_{i}$ is value of the *i*th corresponding fitting point of the received signal.

### 4.3. Waveform Characteristics Analysis

**Figure 7:**
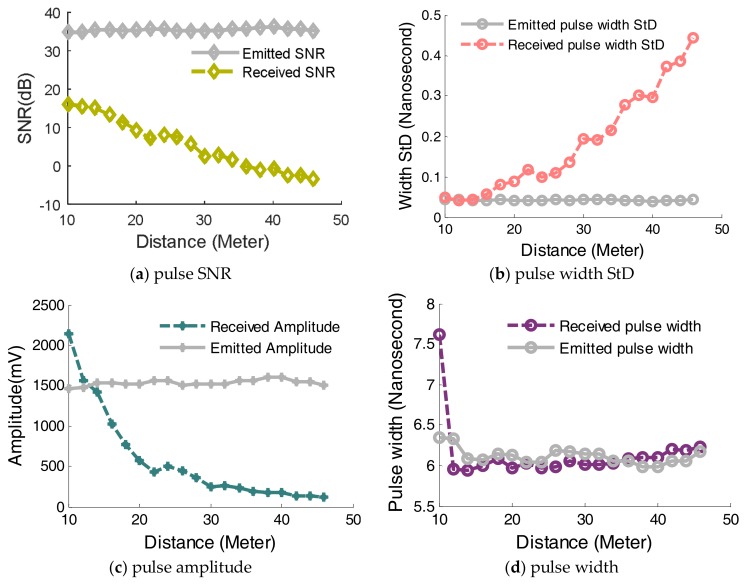
Waveform characteristics varying with range change.

Echo pulses are scattered from the target with background noise, thus a Gaussian function is utilized to smooth the signal and extract the waveform characteristics. Four characteristics of the digitized waveform including signal to noise (SNR), pulse width standard deviation (StD), pulse amplitudes and pulse width are used to demonstrate waveform characteristics as examples, as shown in Figure 7. Seen from Figure 7, it is clear that four characteristics of the emitted pulse keep steady over the entire range, which means that lab-built LiDAR system has stable optical waveform characteristics. Secondly, the received SNR and received amplitude decrease with the increase of the distance while the pulse width StD increases with the increase of the range. This is presumably because the decreased energy of the received pulse can induce the measurement uncertainty of the received pulse width.

### 4.4. Comparison of WR Methods

**Figure 8:**
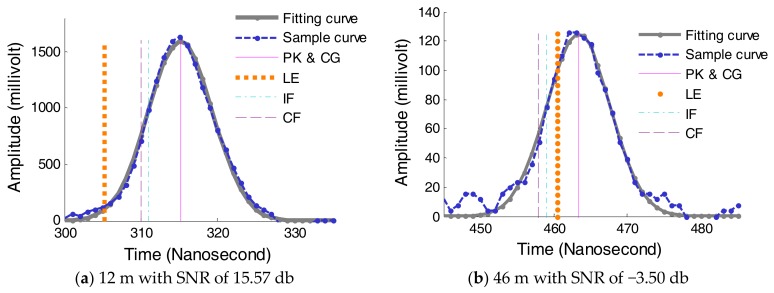
Five time discrimination on received waveform.

The different time discrimination algorithms would result in different range estimation from the emitted and the received waveform, as shown in Figure 8. Time instance is determined by LE method using a threshold of 100 mv, which is affected by the pulse amplitude and background noise. Time instance is determined by CF method of parameters with a delay time of 2 ns and an attenuation of 0.5. Other methods were introduced in Section 3. Seen from Figure 8, it is noted that the amplitude of received pulse decreases with the increase of the distance, because the received energy is inversely proportional to the square of the distance in LiDAR function. Among five methods, the time position on the digitized waveform determined by LE method changes greatly, thus LE method results in larger ranging accuracy. 

**Figure 9:**
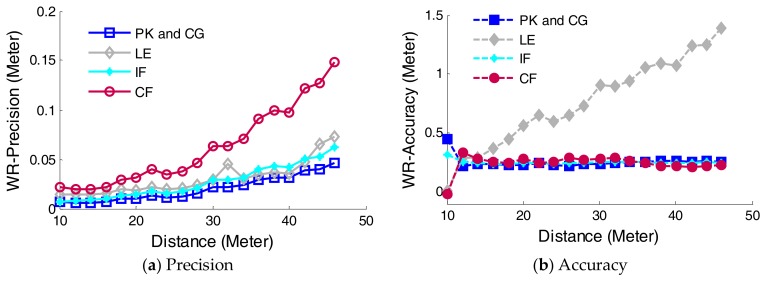
Precision and accuracy comparison of five time discrimination algorithms. PK, CG, LE, IF, and CF stand for peak, center of gravity, leading edge, inflection, and constant fraction.

The ranging precision and accuracy are calculated from 19 groups of experiments (10–46 m), as shown in Figure 9. Two conclusions can be obtained as follows. Firstly, the ranging precision of all waveform-post-processing based time discrimination algorithms decrease with the increase of the distance, among the five methods PK method has the best precision. Secondly, LE method has the worst accuracy than other four discrimination algorithms.

In order to evaluate these five methods of running efficiency, the execution time of the each method is calculated based on CPU processing. All the programs are running on a computer with 4 GB (gigabyte) of RAM (random-access memory), and an Intel(R) Core(TM) i5-6500 CPU @ 3.20 GHz. Because five methods are deduced from different mathematical mechanism, the execution time can be used to denote computational speed of the time instant calculation. Finally, the average execution time of the PK(CG), LE, IF, and CF (peak(center of gravity), leading edge, inflection, and constant fraction) are calculated to be 231.5 ns, 491.0 ns, 285.3 ns, and 365.4 ns for each laser shot, respectively. PK and CG methods run more efficiently than LE, IF, and CF methods because of simpler mathematic expression.

Through analyzing the ranging precision, accuracy and execution time, the PK method can be applied for time discrimination based on waveform-post-processing. Nevertheless, if the asymmetric waveforms are not fitted by Gaussian, the CG method needs more time to calculate the time instance than PK method, thus the PK method can be selected in our further study.

### 4.5. Comparison of PK-WR and AR

**Figure 10:**
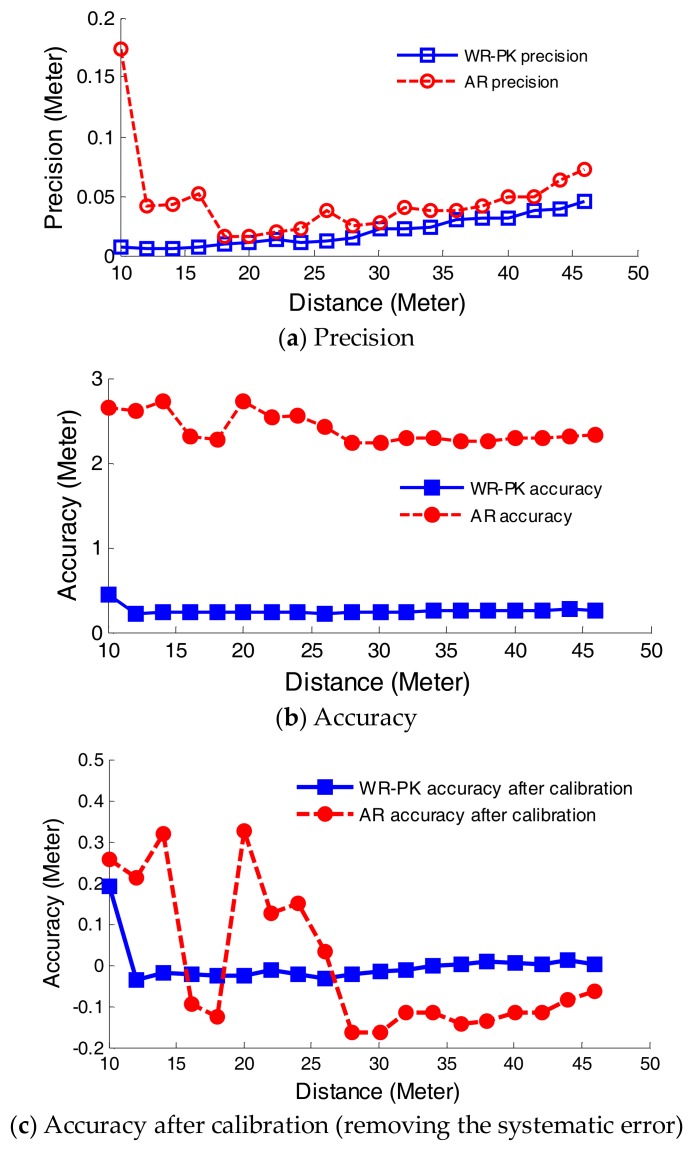
Ranging performances comparison between analog discrete return system (AR) and waveform-digitizing system (WR).

In order to demonstrate the influence of waveform characteristics, the results of analog discrete return system and waveform-digitizing system are compared with each other given the same input signal. The ranging precision and accuracy estimated in two ranging systems are shown in Figure 10. It can be concluded three concerns as follows. Firstly, WR precision based on PK method is better than AR precision based on analog discrete return system. Secondly, at short-distance detection (within 18 m) the AR precision decreases with the increase of the range, but the WR precision increases with the increase of the range, presumably because the energy of echo pulses at short-distance is close to saturation or the waveform distortion caused by the limited received bandwidth, which results in a larger ranging error in the constant fraction discriminator of the analog discrete return system. Thirdly, at long-distance (far from 19 m) the precision values of both ranging systems increase with the increase of the range but stability of the WR precision is better than that of AR precision. Fourthly, the accuracy of AR data is worse than that of WR data. The ranging errors of both ranging systems include systematic and random, which can be obtained from results of the measured data minus a true value. After removing the systematic error, the random error of AR method is larger than that of WR method, as shown in Figure 10c. In conclusion, the accuracy and the precision of the method based on waveform-post-processing are superior to these of analog discrete return system.

From the definition in Section 4.2 and Figure 10, two indicators including bias and nonlinearity are calculated to evaluate the ranging accuracy. The biases of the WR-PK accuracy and AR accuracy are 25.48 cm and 24.00 cm, respectively. The nonlinearity of the WR-PK accuracy and AR accuracy are 4.74 cm and 12.53 cm, respectively. The above results show that the bias and nonlinearity of the WR-PK method is less than AR method. Furthermore, the accuracy of WR-PK and AR method are calculated after calibration, whose maximum values are 19.44 cm and 32.72 cm, and mean values are 2.43 cm and 15.11 cm, respectively. In order to give a specification of the WR-PK and AR method, their precision is given the maximum precision value over the entire measurement range and their accuracy is given the maximum calibration accuracy value over the entire measurement range. The specification is present in Table 1.

### 4.6. Relationship between Waveform Characteristics and Ranging Precision

In order to analyze the relationship between the waveform characteristics and the ranging precision, the maximal information coefficient (MIC) method [17] is employed to calculate strength of their relation. There are three parameters—MIC (0 ≤ MIC ≤ 1), MIC-ρ2 (from −1 to 1), and MAS (0 ≤ MAS ≤ 1)—that can be used for analyzing the influence of waveform characteristics. MIC captures a wide range of associations and provides a score for identifying relationships and roughly equal to R2 on functional relationships. MIC assigns scores that tend to 0 to statistically independent variables. MIC-ρ2 can be also defined a natural measure of nonlinearity. Here, ρ denotes the Pearson product-moment correlation coefficient, a measure of linear dependence. The statistic MIC-ρ2 is near 0 for linear relationships and large for nonlinear relationships with high values of MIC. The maximum asymmetry score (MAS) detected deviation from monotonicity for detecting periodic relationships with unknown frequencies that vary over time. In the following, the result of the relationship between waveform characteristics (amplitude, SNR, pulse width, and pulse width StD) and ranging precision is presented in Table 2.

**Figure 11:**
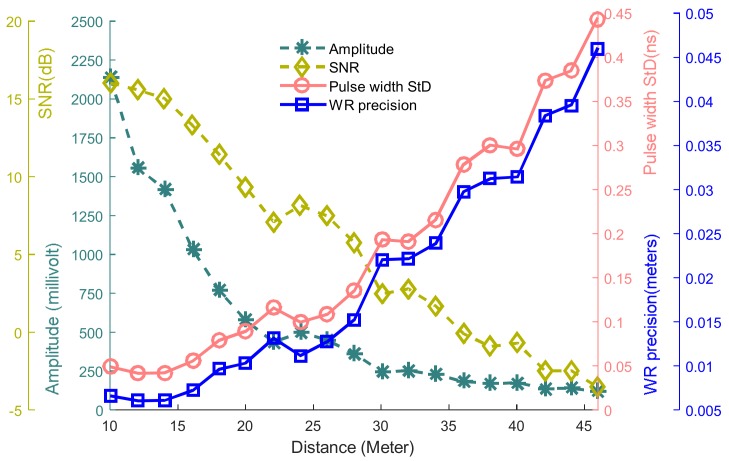
Waveform characteristics influencing on ranging precision.

Seen from Figure 11 and Table 2, the relationship between the WR-PK precision and other three characteristics of waveform including the amplitude, SNR and pulse width StD were analyzed respectively. Pulse width StD has a high linear relatedness relationship with PK-WR precision because it has a MIC close to 1 and MIC-ρ2 close to 0. Amplitude has a nonlinear relatedness relationship with WR-PK precision because they have MIC close to 1 and MIC-ρ2 close to 0.47. SNR should be close to linear relatedness relationship. The same result will be concluded for the relationship between the PK precision and the received SNR. Because of that, the SNR of ranging system needed to be improved to achieve a high precision in long-distance detection. MAS calculated from all factors are equal to 0, which indicates that PK-WR precision has a good monotonicity relative to three factors.

### 4.7. Discussion

This manuscript employs a waveform-post-processing based time discrimination algorithms (WR-PK method) to measure distance which shows the excellent ranging performance of accuracy and precision by comparison with the results obtained from analog discrete return system (AR results). Among four waveform-post-processing based time discrimination algorithms (WR), WR-PK has the better performance than the other three methods (WR-TH, WR-CF, and WR-IF) because of its lower execution time, steady accuracy, and high precision. The WR-CG method could be not employed because it costs more time and low ranging accuracy when it is applied on asymmetric echo waveforms. In future study, an efficient time discrimination method will run in FPGA-based real-time processing system, thus the execution time is a crucial criteria for improving FPGA running efficiency and reducing FPGA resource in the implementation of time discrimination algorithms. 

In order to evaluate the WR-PK method, a comparison experiment is carried out between analog discrete return system (AR data) and waveform-digitizing system (WR data). The results show that WR-PK method has the better accuracy and precision than AR results. It can be explained by three reasons: firstly, the signal conditioning circuits including AGC and CFD exit heavy time delay that will worsen the accuracy much more. Secondly, the limited bandwidth of the signal conditioning circuit can change the shape of received waveform, thus the ranging precision would be worsen because CFD is sensitive to the input waveform. WR-PK method has the smallest error and the best ranging accuracy. Thirdly, AR ranging precision is limited by resolution of TDC chip (45 ps) and cannot be further improved through other methods, contrarily, WR ranging precision is mainly limited by the sample rate of ADC but can be improved to sub-sample-rate precision through Gaussian fitting. In summary, the WR-PK method can increase the accuracy and precision of LiDAR ranging. 

WR-PK precision has related with the three characteristics of the received waveform, including the amplitude, SNR, and received pulse width StD. The received pulse width StD has a high linear relationship with the ranging precision, thus ensuring that the received pulse width will keep a steady level for measuring a constant distance is a very important work. Besides, improving SNR can achieve a high precision in long distance ranging.

## 5. Conclusions

This paper studies the ranging performance comparison between the waveform based time discrimination methods and analog discrete return system. A peak detection method (WR-PK) in the waveform-post-processing has a low execution time (231.5 ns), high ranging accuracy (2.43 cm mean accuracy), and stable precision (2.01 cm mean precision and 4.59 cm maximum precision). We will use a FPGA-based real-time processing system and implement the algorithms for time discrimination method based waveform, thus the runtime of ranging measurement on-line needs to be considered for large amounts of point-cloud data. Using a novel statistic mathematical method (MIC), WR-PK precision has a high linear relationship with the received pulse width StD. Thus keeping received pulse width as stable as possible for improving the ranging precision is worthy of further investigation. There are some defects of using Gaussian functions to determine the time of transmitted and received pulses under varied situations. Algorithms of processing received pulse with complex models will be researched. Influences of other waveform characteristics on LiDAR ranging accuracy and precision will also be studied in the future. 

## Figures and Tables

**Table 1:** The ranging accuracy and precision.

Methods	WR-PK	AR
Maximum precision (m)	0.0459	0.1732
Mean precision (m)	0.0201	0.0457
Maximum accuracy after calibration (m)	0.1944	0.3272
Mean accuracy after calibration (m)	0.0243	0.1511

**Table 2:** The MIC, MIC-ρ2, MAS evaluation results.

X Variables	Y Variables	MIC (Strength)	MIC-ρ2 (Nonlinearity)	MAS (Non-Monotonicity)
PK-WR precision	Amplitude	0.998	0.4704616	0
PK-WR precision	SNR	0.998	0.10022944	0
PK-WR precision	Pulse width StD	0.998	−0.00042	0
PK-WR precision	Pulse width	0.46476	0.4613367	0
